# Orbital apex syndrome secondary to a fungal nasal septal abscess caused by *Scedosporium apiospermum* in a patient with uncontrolled diabetes: a case report

**DOI:** 10.1186/s12879-017-2753-6

**Published:** 2017-09-26

**Authors:** Ippei Kishimoto, Shogo Shinohara, Tetsuhiro Ueda, Shoichi Tani, Hajime Yoshimura, Yukihiro Imai

**Affiliations:** 1 Department of Otolaryngology, Head and Neck Surgery, Graduate School of Medicine, Kyoto University, 54 Shogoin Kawahara-cho, Sakyo-ku, Kyoto, 606-8507 Japan; 2 Department of Otolaryngology – Head and Neck Surgery, Kobe City Medical Center General Hospital, 2-1-1 Minatojima-Minamimachi, Chuo-ku, Kobe, 650-0047 Japan; 3 Department of Neurology, Kobe City Medical Center General Hospital, 2-1-1 Minatojima-Minamimachi, Chuo-ku, Kobe, 650-0047 Japan; 4 Department of Neurosurgery, Kobe City Medical Center General Hospital, 2-1-1 Minatojima-Minamimachi, Chuo-ku, Kobe, 650-0047 Japan; 5 Department of Clinical Pathology, Kobe City Medical Center General Hospital, 2-1-1 Minatojima-Minamimachi, Chuo-ku, Kobe, 650-0047 Japan

**Keywords:** *Scedosporium apiospermum*, *Pseudallescheria boydii*, Nasal septal abscess, Orbital apex syndrome, Endoscopic sinus surgery

## Abstract

**Background:**

Orbital apex syndrome is a localized type of orbital cellulitis, where mass lesions occur at the apex of the cranial nerves. Although nasal septal abscess is uncommon, the organism most likely to cause nasal septal abscess is *Staphylococcus aureus*, and fungal septal abscesses are rare. Here we present an extremely rare and serious case of orbital apex syndrome secondary to fungal nasal septal abscess caused by *Scedosporium apiospermum* in a patient with uncontrolled diabetes.

**Case presentation:**

A 59-year-old man with a 1-month history of headache underwent consultation in an otolaryngological clinic of a general hospital. He was diagnosed with nasal septal abscess and was treated with incisional drainage and 1 month of an antibiotic drip; however, his symptoms persisted. The patient later complained of diplopia due to bilateral abducens nerve palsy, and was then referred to the department of Otolaryngology – Head and Neck Surgery, Kobe City Medical Center General Hospital. The septal lesion was biopsied under general anesthesia, and *S. apiospermum* was detected using polymerase chain reaction. The patient was treated with an antifungal drug and surgical resection of the lesion was performed. Although the patient survived, he lost his eyesight.

**Conclusions:**

This patient represents the second reported case of nasal septal abscess and orbital apex syndrome caused by *S. apiospermum*. If not treated properly, septal abscess can be life-threatening and cause severe complications, such as ablepsia.

## Background

Nasal septal abscess is a rare complication, and is defined as collection of purulent material between the cartilaginous or bony septum and the mucoperichondrium or mucoperiosteum [1]. The most common organism responsible for nasal septal abscess is *Staphylococcus aureus*, followed by *Streptococcus pneumoniae* and *S. viridans* [1, 2]. Fungal septal abscess is rare. *Scedosporium apiospermum* is a ubiquitous filamentous fungus that is present in soil, sewage, and polluted waters [3]. To date, there has been only one report on nasal septal abscess caused by *S. apiospermum* [4], and one report on orbital apex syndrome caused by *S. apiospermum* [5]. Here we report an extremely rare case of orbital apex syndrome secondary to fungal septal abscess caused by *S. apiospermum* in a patient with uncontrolled diabetes.

## Case presentation

**Fig. 1:**
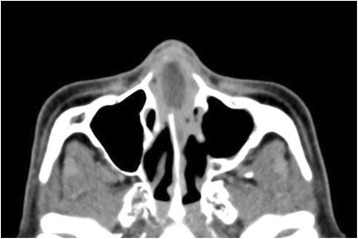
Axial view of contrast-enhanced computed tomography (CT). A swollen, ring-enhancing lesion of the anterior nasal septum was observed

A 59-year-old man with a 1-month history of headache was examined in an otolaryngological clinic of a general hospital. The patient’s anterior nasal septum was swollen, and he was diagnosed with nasal septal abscess and admitted to the hospital the same day. Enhanced computed tomography (CT) revealed a swollen, ring-enhanced lesion in the anterior nasal septum (Fig. 1). He was treated with incisional drainage of the abscess and antibiotic drip for 1 month (fosfomycin, ampicillin/sulbactam, ceftriaxone, and meropenem). In addition, insulin-based therapy was started because the patient had uncontrolled diabetes and hemoglobin A1c of 13.3%. His symptoms persisted despite 1 month of antibiotic drip. Pus from the nasal septum was cultured three times. Culture tests detected low concentrations of *Capnocytophaga spp*. and *Serratia marcescens*, which were sensitive to the antibiotic drip. No fungi were detected. The septal lesion was biopsied twice and showed granulation-like tissue with infiltrated inflammatory cells (nothing indicating malignancy or fungus). Despite the antibiotic drip for >1 month, diplopia developed due to bilateral abducens nerve palsy, with subsequent impairment of right eye movement and eventual visual loss. The patient was then referred to our department.

The patient’s initial visit revealed a saddle nose. Nasal fiberscope revealed bulging of the nasal septum and granulation-like tissue on the right side of the nasal septum. The patient’s left eye fixed at the central position and the supraduction and adduction of the right eye were limited. His visual acuities were light perception in the right eye and “count fingers” in the left eye. The functions of the 5th, 7th, and 8–12th cranial nerves were intact.

**Fig. 2:**
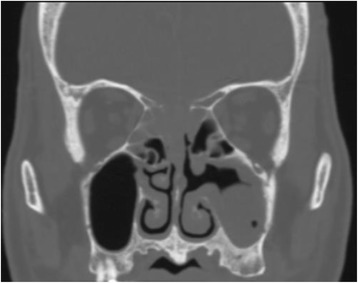
Coronal view of non-contrast-enhanced CT. A poorly marginated lesion with soft tissue density was observed in the superior nasal septum, invading the bone in the anterior cranial fossa. A mass with soft tissue density is apparent in the left maxillary sinus

**Fig. 3:**
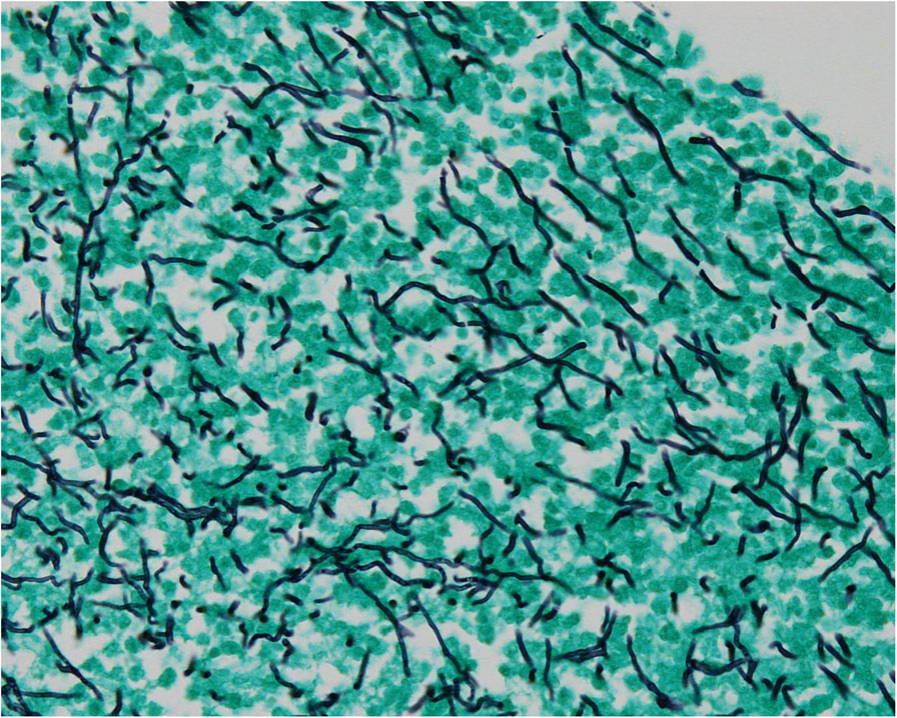
Histopathological findings of the ball-like lesion in the left maxillary sinus. These fungi have slightly thinner filaments than those of *Aspergillus spp*. They are consistent with characteristics of *Scedosporium apiospermum*. (Grocott’s methenamine silver stain, ×40)

**Fig. 4:**
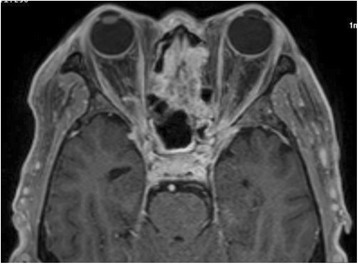
Contrast-enhanced magnetic resonance imaging (MRI), axial view. A strongly enhanced lesion was observed in the nasal septum, ethmoid sinuses, bilateral orbital apexes, and cavernous sinus

The patient’s hemoglobin A1c was 9.2%. Plain CT showed a poorly marginated lesion with soft tissue density at the superior nasal septum, which was invading the anterior cranial fossa. Another mass with soft tissue density appeared in the left maxillary sinus (Fig. 2). A spinal tap and cerebrospinal fluid culture were immediately performed, and the results revealed elevated glucose levels (130 mg/dL) and cell counts (mononucleosis 19/m^3^, polynucleosis 1/m^3^). No pathogen was detected by microscopic examination or culture methods. Owing to the possibility of a fungal and/or bacterial infection, we prescribed an antibiotic (ampicillin/sulbactam) and antifungal (amphotericin B) drip. Subsequently, we performed diagnostic endoscopic sinus surgery under general anesthesia. Histopathological examination revealed that the ball-like lesion in the left maxillary sinus harbored fungi, with filaments slightly thinner than those of *Aspergillus spp.* (Fig. 3). A commercially available fungal DNA detection kit (Robust HotStart ReadyMix PCR Kit; KAPA biosystems, Inc., U.S.A.) was used to detect pathogens in tissues from the nasal septal lesion and ball-like fungal lesion in the left maxillary sinus. A broad-range polymerase chain reaction (PCR) was performed targeting the highly conserved sequence of the ribosomal RNA genes in fungal DNA shared by most fungi. Internal transcribed spacer (ITS) regions in ribosomal DNA were then amplified by PCR using primers ITS1F (5′-TCCGTAGGTGAACCTGCGG-3′) and ITS4R (5′-TCCTCCGCTTATTGATATGC-3′). PCR products of 150–500 bp were obtained and sequenced to match a known DNA sequence from a reference database (BLAST: Basic Local Alignment Search Tool). The organism detected by PCR and tissue cultures was *S. apiospermum*. Subsequent enhanced magnetic resonance imaging (MRI) revealed strongly enhanced lesions of the nasal septum, ethmoid sinuses, bilateral orbital apexes, and cavernous sinus. The dura along the anterior and bilateral middle cranial fossa was thickened and well-enhanced. (Fig. 4). The diagnosis was orbital apex syndrome and hypertrophic pachymeningitis secondary to fungal nasal septal abscess caused by *S. apiospermum*.

Drug susceptibility testing (Table 1) led us to change the antifungal drip to voriconazole (500 mg/day). Since voriconazole had no apparent effect on the patient’s symptoms after four days, we surgically resected the lesion under general anesthesia.

**Table 1:** Drug susceptibility testing using the Clinical and Laboratory Standards Institute M38-A2

Antifungal drugs	Abbreviation	MIC
		(μg/ml)
Flucytosine	5FC	>64
Amphotericin-B	AMPH	4
Miconazole	MCZ	1
Fluconazole	FLCZ	32
Itraconazole	ITCZ	1
Micafungin	MCFG	>16
Voriconazole	VRCZ	0.5

*MIC* minimum inhibitory concentration

Using endoscopy-assisted open skull base surgery, we removed the fungal ball-like lesion and the mucosa of the left maxillary sinus. We then incised the anterior border of the nasal septum and removed the mucosa of the bilateral ethmoid and sphenoid sinuses. After anterior craniotomy, we resected the anterior skull base between both sides of the ethmoid tegmen, from the frontal table anteriorly to the optic chiasm posteriorly. We avoided the extirpation of the eye ball or removal of the orbital content, because the medial bony wall of the orbit was intact.

The resected nasal septum was thickened and necrotic, strongly suggesting infectious inflammation and necrosis of the tissue. Mild mucosal edema was observed in the bilateral ethmoid and sphenoid sinuses. No fungal invasion or pus was observed. The dura was thinned just above the affected part of the cranial fossa, suggesting that the infection and inflammation had spread to the dura; however, there was no apparent dural deficit, and the dura was preserved. Following resection, we reconstructed the affected anterior cranial fossa with pericranium.

**Fig. 5:**
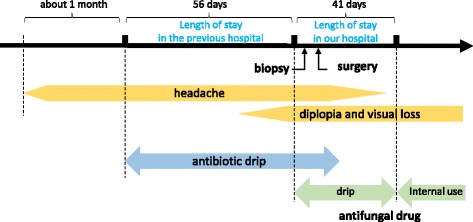
The timeline of the patient’s illness. The patient visited the previous hospital 1 month after the onset of symptoms. In spite of the treatments including incisional drainage of the nasal abscess and antibiotic drip for two months, the symptoms didn’t improve. After surgical resection of the lesion, the patient recovered with sequela such as diplopia and visual loss

The patient’s headache decreased and almost disappeared 3 weeks postoperatively. His preoperative mild fever and inflammation normalized, and he was discharged on postoperative day 29. The patient continued oral voriconazole (400 mg/day) until 12 months post-surgery, when bilateral ocular movements completely improved; however, loss of visual acuity persisted. At 22 months post-surgery, a follow-up MRI demonstrated that the enhanced lesion in the orbital apex diminished. Thirty months has passed without any evidence of recurrence until now. The timeline of the patient’s illness is illustrated in Fig. 5.

## Discussion

Nasal septal abscess is curable without life-threatening complications, if it is promptly diagnosed and appropriately treated. However, late diagnosis and treatment is associated with serious complications, such as meningitis [6], intracranial sinus venous thrombosis [7], or even death [8]. Early diagnosis and immediate therapy is important to avoid a cosmetic nasal deformity or intracranial infection [9]. Serious complications of nasal septal abscesses may occur, especially in immunosuppressed patients [10]. In our case, despite several cultures and biopsies, the causative agent had not been identified. Approximately 2 months elapsed before *S. apiospermum* was identified as the causative agent. Owing to this delay, infectious inflammation from the abscess had spread superiorly, resulting in orbital apex syndrome. Although the patient survived because of treatment with an antifungal drip and a surgical procedure, he lost sight in both eyes.

The most common organism responsible for nasal sepal abscess is *S. aureus* [1]. Others include *S. pneumoniae*, *S. viridans*, *S. epidermidis*, and *Haemophilus influenzae* [11, 12]. Although fungi rarely cause nasal septal abscess, its possibility should be considered, especially in immunocompromised patients.

*S. apiospermum* is a ubiquitous filamentous fungus that is present in soil, sewage, and polluted water [3]. The common clinical manifestations of *S. apiospermum* infection are mycetomas, involving cutaneous and subcutaneous tissues, fascia, joints, and bones; opportunistic infections in immunocompromised patients; and non-opportunistic pulmonary infection, especially in patients with respiratory compromise, such as cystic fibrosis, bronchiectasis, or chronic obstructive pulmonary disease [3].

Disseminated *S. apiospermum* infection, which carries a poor prognosis, is usually observed in immunocompromised patients, although it has also been reported in immunocompetent patients [3]. Reports of serious infections caused by this fungus are increasing [3]. Sinusitis caused by *S. apiospermum* may appear in immunocompetent or immunocompromised patients [3].

Previously, *S. apiospermum* was reported to cause nasal septal abscess in only one case [4]. Thus, our patient is only the second reported case with nasal septal abscess caused by *S. apiospermum*. In the first case, the patient was treated with oral steroids for uncontrolled asthma, whereas our patient had uncontrolled diabetes. In both cases, the patients were immunocompromised, which is considered to be a major factor for developing *S. apiospermum* infection. In addition, our patient is only the second reported case of *S. apiospermum*-related orbital apex syndrome. The first case was that of orbital apex syndrome secondary to sphenoidal sinus mycetoma caused by *Pseudallescheria boydii* in a 92-year-old immunocompetent patient [5]. Therefore, *S. apiospermum* should be considered to be a causative agent of infections in both immunocompetent and immunocompromised patients.

Treatment of infections caused by *Scedosporium spp.* is especially challenging because of their resistance to antifungal drugs [3]. Most *S. apiospermum* fungi are resistant to amphotericin B, caspofungin, anidulafungin, isavuconazole, and itraconazole [13]. Among “azoles,” voriconazole and posaconazole demonstrate the highest in vitro activity, and micafungin is the most active echinocandin antifungal drug [13]. Surgical excision of lesions has been a part of the standard care of patients with *S. apiospermum* infection, and should be considered whenever possible, even among immunocompetent patients [3]. When treating a nasal septal abscess, serious complications could be prevented by prompt, effective surgery [14]. In the present case, along with the antifungal drugs, the surgical treatment was vital because the nasal septal abscess had worsened and was already leading to the development of orbital apex syndrome when the patient was first examined by us. During surgery, we removed the infected tissue, including the necrotic nasal septum, inflammatory sinus mucosa, and the bone in the anterior cranial fossa. The orbit and inflammatory dura were preserved. Since orbital apex syndrome was diagnosed and intraorbital spread of inflammation was suspected, we considered exenteration of the orbit to be necessary. We preserved the orbit’s content but removed the bony wall on the optic chiasm which appeared necrotic. During surgical treatment of acute, invasive fungal sinusitis, the decision to proceed with orbital exenteration should be carefully considered because it may not favor the patient’s overall chance for survival [15].

## Conclusion

We present a rare case of orbital apex syndrome secondary to a fungal nasal septal abscess caused by *S. apiospermum* in a patient with uncontrolled diabetes. After treatment with an antifungal drug and surgical resection, the patient survived but lost his eyesight. Prompt diagnoses of fungal infections are often difficult. Therefore, early consideration of the possibility of a fungal pathogen as the causative agent is important. Similarly, appropriate treatment as well as repeated diagnostic examinations, including PCR, fungal cultures, and histopathological analysis should be promptly conducted in order to obtain favorable outcomes and increase the patients’ chances of survival.

## Abbreviations

CT: Computed tomography
ITS: Internal transcribed spacer
MRI: Magnetic resonance imaging
PCR: Polymerase chain reaction

## References

[1] Ambrus PS, Eavey RD, Baker AS, Wilson WR, Kelly JH (1981). Management of nasal septal abscess. Laryngoscope.

[2] Canty PA, Berkowitz RG (1996). Hematoma and abscess of the nasal septum in children. Arch Otolaryngol Head Neck Surg.

[3] Cortez KJ, Roilides E, Quiroz-Telles F, Meletiadis J, Antachopoulos C, Knudsen T (2008). Infections caused by Scedosporium spp. Clin Microbiol Rev.

[4] Patel R, Orlandi RR (2015). Fungal septal abscess complicating maxillary sinus fungus balls in an immunocompetent host. Allergy Rhinol (Providence).

[5] Thiagalingam S, Fernando GT, Tan K, O'Donnell BA, Weeks K, Branley M (2004). Orbital apex syndrome secondary to Pseudallescheria boydii fungal sinusitis in an immunocompetent patient. Clin Exp Ophthalmol.

[6] Eavey RD, Malekzakeh M, Wright HT (1977). Bacterial meningitis secondary to abscess of the nasal septum. Pediatrics.

[7] Frank GS, Smith JM, Davies BW, Mirsky DM, Hink EM, Durairaj VD (2015). Ophthalmic manifestations and outcomes after cavernous sinus thrombosis in children. J AAPOS.

[8] Shah SB, Murr AH, Lee KC (2000). Nontraumatic nasal septal abscesses in the immunocompromised: etiology, recognition, treatment, and sequelae. Am J Rhinol.

[9] Jalaludin MA (1993). Nasal septal abscess-retrospective analysis of 14 cases from University Hospital, Kuala Lumpur. Singapore Med J.

[10] Debnam JM, Gillenwater AM, Ginsberg LE (2007). Nasal septal abscess in patients with immunosuppression. AJNR Am J Neuroradiol.

[11] Pang KP, Sethi DS (2002). Nasal septal abscess: an unusual complication of acute spheno-ethmoiditis. J Laryngol Otol.

[12] Ehrlich A (1993). Nasal septal abscess: an unusual complication of nasal trauma. Am J Emerg Med.

[13] Lackner M, Fernandez-Silva F, Guarro J, Lass-Florl C (2014). Assessing micafungin/triazole combinations for the treatment of invasive scedosporiosis due to Scedosporium apiospermum and Scedosporium boydii. J Antimicrob Chemother.

[14] Dinesh R, Avatar S, Haron A, Suhana A (2011). Nasal septal abscess with uncontrolled diabetes mellitus: case reports. Med J Malaysia.

[15] Turner JH, Soudry E, Nayak JV, Hwang PH (2013). Survival outcomes in acute invasive fungal sinusitis: a systematic review and quantitative synthesis of published evidence. Laryngoscope.

